# Vasculitis as a Major Morbidity Factor in Patients With Partial RAG Deficiency

**DOI:** 10.3389/fimmu.2020.574738

**Published:** 2020-10-21

**Authors:** Christoph B. Geier, Jocelyn R. Farmer, Zsofia Foldvari, Boglarka Ujhazi, Jolanda Steininger, John W. Sleasman, Suhag Parikh, Meredith A. Dilley, Sung-Yun Pai, Lauren Henderson, Melissa Hazen, Benedicte Neven, Despina Moshous, Svetlana O. Sharapova, Snezhina Mihailova, Petya Yankova, Elisaveta Naumova, Seza Özen, Kevin Byram, James Fernandez, Hermann M. Wolf, Martha M. Eibl, Luigi D. Notarangelo, Leonard H. Calabrese, Jolan E. Walter

**Affiliations:** ^1^Immunology Outpatient Clinic, Vienna, Austria; ^2^Harvard Medical School, Massachusetts General Hospital, Boston, MA, United States; ^3^Department of Cancer Immunology, Institute for Cancer Research, Oslo University Hospital, Radiumhospitalet, Oslo, Norway; ^4^University of South Florida and Johns Hopkins All Children's Hospital, Saint Petersburg, FL, United States; ^5^Division of Allergy, Immunology and Pulmonary Medicine, Duke University School of Medicine, Durham, NC, United States; ^6^Emory University School of Medicine, Atlanta, GA, United States; ^7^Department of Immunology, Harvard Medical School, Boston Children's Hospital, Boston, MA, United States; ^8^Division of Hematology-Oncology, Harvard Medical School, Boston Children's Hospital, Boston, MA, United States; ^9^Department of Pediatric Oncology, Dana-Farber Cancer Institute, Boston, MA, United States; ^10^Harvard Medical School, Boston, MA, United States; ^11^Division of Immunology, Department of Rheumatology, Boston Children's Hospital, Boston, MA, United States; ^12^Imagine Institute, Paris Descartes-Sorbonne Paris Cité University, Paris, France; ^13^Pediatric Hematology-Immunology and Rheumatology Unit, Necker-Enfants Malades University Hospital, Assistance Publique-Hôpitaux de Paris, Paris, France; ^14^Laboratory “Immunogenetics of Pediatric autoimmune diseases”, INSERM UMR1163, Institut Imagine, Université Paris Descartes Sorbonne Paris Cité, Paris, France; ^15^Laboratory of Genome Dynamics in The Immune System, Paris, France; ^16^Research Department, Belarusian Research Center for Pediatric Oncology, Hematology and Immunology, Minsk, Belarus; ^17^Department of Clinical Immunology Medical University of Sofia, Sofia, Bulgaria; ^18^Division of Rheumatology, Department of Pediatrics, Hacettepe University Faculty of Medicine, Ankara, Turkey; ^19^Cleveland Clinic Center for Vasculitis Care and Research, Cleveland, OH, United States; ^20^Sigmund Freud Private University- Medical School, Vienna, Austria; ^21^Biomedizinische Forschungs GmbH, Vienna, Austria; ^22^Laboratory of Clinical Immunology and Microbiology, NIAID, National Institutes of Health, Bethesda, MD, United States; ^23^University of South Florida at Johns Hopkins All Children's Hospital, Saint Petersburg, FL, United States; ^24^Division of Allergy and Immunology, Massachusetts General Hospital for Children, Boston, MA, United States

**Keywords:** vasculitis, primary immumunodeficiencies, rag deficiency, severe combined immunodeficiencies (SCID), autoimmunity, combined immunodeficiency with granuloma and/or autoimmunity, atypical SCID

## Abstract

Vasculitis can be a life-threatening complication associated with high mortality and morbidity among patients with primary immunodeficiencies (PIDs), including variants of severe and combined immunodeficiencies ((S)CID). Our understanding of vasculitis in partial defects in recombination activating gene (RAG) deficiency, a prototype of (S)CIDs, is limited with no published systematic evaluation of diagnostic and therapeutic modalities. In this report, we sought to establish the clinical, laboratory features, and treatment outcome of patients with vasculitis due to partial RAG deficiency. Vasculitis was a major complication in eight (13%) of 62 patients in our cohort with partial RAG deficiency with features of infections and immune dysregulation. Vasculitis occurred early in life, often as first sign of disease (50%) and was complicated by significant end organ damage. Viral infections often preceded the onset of predominately non-granulomatous-small vessel vasculitis. Autoantibodies against cytokines (IFN-α, -ω, and IL-12) were detected in a large fraction of the cases tested (80%), whereas the majority of patients were anti-neutrophil cytoplasmic antibodies (ANCA) negative (>80%). Genetic diagnosis of RAG deficiency was delayed up to 2 years from the onset of vasculitis. Clinical cases with sole skin manifestation responded well to first-line steroid treatment, whereas systemic vasculitis with severe end-organ complications required second-line immunosuppression and/or hematopoietic stem cell transplantation (HSCT) for definitive management. In conclusion, our data suggest that vasculitis in partial RAG deficiency is prevalent among patients with partial RAG deficiency and is associated with high morbidity. Therefore, partial RAG deficiency should be included in the differential diagnosis of patients with early-onset systemic vasculitis. Diagnostic serology may be misleading with ANCA negative findings, and search for conventional autoantibodies should be extended to include those targeting cytokines.

## Introduction

The recombination-activating gene 1 (*RAG1*) and *RAG2* encode lymphoid-specific proteins that are essential for V(D)J recombination, promoting diversification the T and B cell repertoire (TCR, BCR), and receptor editing (1, 2). First described in 1996 by Schwarz et al. null mutations in the *RAG1/RAG2* genes result in T- and B-cell-negative SCID (3). The spectrum of the disease was soon extended to include patients with Omenn syndrome and leaky SCID (LS), with relative recombinase activity lower than 5% resulting in the generation of restricted, oligoclonal lymphocytes that are enriched for self-reactive specificities (3, 4). Hypomorphic variants with more preserved relative recombinase activity (in average 5–30%), compared to OS and LS, result in a broader spectrum of distinct phenotypes, including, combined immunodeficiency with granuloma and/or autoimmunity (CID-G/A) (5, 6), primary antibody deficiencies (7–9), idiopathic CD4^+^ T cell lymphopenia (10), hyper-IgM syndrome (11), and sterile chronic multifocal osteomyelitis (12). This highly vulnerable patient population presents with a variety of autoimmune and hyperinflammatory complications including refractory cytopenias (84.1%), granulomas (23.8%), and inflammatory skin disorders (19.0%) (13).

Vasculitis is observed in various chronic diseases; it is characterized by inflammation of blood vessels, and is classified into large, medium, and small vessel vasculitis, based on the diameter of the affected vessels. While the inflammatory process may be confined to a single organ or site, it may also involve several organ systems, resulting in a vast variety of clinical presentations. Although the specific pathogenesis has yet to be identified, most vasculitides have complex etiology, and both genetic and environmental factors appear to contribute to the pathogenesis (14). In recent years, vasculitis has been described as a feature of various forms of PID, including those with pathogenic STAT1 gain-of-function variants, adenosine deaminase 2 (ADA2) deficiency, X-linked lymphoproliferative syndrome (XLP) type 1, Wiskott-Aldrich-syndrome (WAS), TAP1/2 deficiency, complement deficiency, and NOD2 deficiency (15–21).

Systemic vasculitis has been described as severe complication with significant end-organ damage in patients with partial RAG deficiency (pRD) (13). However, our understanding of vasculitis in RAG deficiency is limited, with no published systematic evaluation of clinical evolution, diagnostic, and therapeutic modalities. Herein we sought to describe the clinical, laboratory features, and treatment outcome of patients with vasculitis due to pRD.

## Materials and Methods

We maintain a curated patient database (IRB protocol #Pro00025693) of 62 cases of RAG deficiency with autoimmune/hyperinflammatory complications from which we collected the following information: sex, age (current as of March 2020, at clinical diagnosis of immunodeficiency and/or autoimmunity, at molecular diagnosis of RAG deficiency, and at death or HSCT), genotype (specific *RAG1 or RAG2* variants), immune phenotype (lymphocyte counts and function, immunoglobulin levels, and autoantibodies), vasculitis (type, age at onset, preceding infections if available, length, and severity), other autoimmune/hyperinflammatory complications, and therapies trialed (including response and complications) (13). This database is continuously updated with relevant cases following literature search and/or personal communication. Patients with vasculitis were identified from our curated database and physicians were individually contacted for additional details. All patients remained deidentified and were previously consented locally. A structured datasheet was utilized to collect clinical information from the treating physician. All patients were assigned as CID-G/A based on published criteria by Delmonte et al. (22). Although we do acknowledge, that currently CID-G/A has not been fully defined by either the Primary Immunodeficiency Consortium (PIDTC) or the Expert Committee of International Union of Immunodeficiency Societies (IUIS) (23). In Table 1 we provide detailed clinical information on patients with vasculitis and pRD. Predicted relative V(D)J recombination activity was recorded as previously described (24, 25). Lymphocyte panel and immunoglobulin levels were determined by clinical laboratory testing at the patient's home institution. Similarly, ANCA and antinuclear antibodies (ANA) were detected by indirect immunofluorescence assay, other autoantibodies were detected by Enzyme-Linked Immunosorbent Assay (ELISA) as part of the routine medical care (6, 9, 26–28). Anti-cytokine antibodies were detected by ELISA as previously described (29). For phenotypic description, healthy age matched blood donors (*n* = 25), and RAG deficient patients with similar clinical phenotypes (CID-G/A and atypical SCID, *n* = 48) served as healthy and disease controls. Statistical comparisons were performed by calculating the Mann Whitney *U*-test using Prism Graphpad 8.4 software. Statistically significant differences obtained in intergroup comparisons were confirmed by Kruskal–Wallis one-way analysis of variance using Prism Graphpad 8.4 software. Kaplan-Meier curves were compared using a log-rank (Mantel-Cox) test. Values of *p* < 0,05 were considered as significant (ns statistically not significant, ^*^p ≤ 0.05, ^**^p ≤ 0.01, ^***^p ≤ 0.001, ^****^p ≤ 0.0001).

**Table 1:** Detailed clinical information on patients with vasculitis due to RAG deficiency.

**ID**	**Age**	**Gene**	**Mutation**	**RAG activity**	**RAG phenotype**	**Type of vasculitis**	**Severity of vasculitis**	**Age at onset of vasculitis**	**Diagnosis**	**Autoantibodies**	**Other Autoimmunity**	**Treatment**			**Respose to therapy**	**Overall outcome (cause of death)**	**Reference**
												**First-Line**	**Second-Line**	**Third-Line**			
Patient 1	6 yrs	*RAG1*	a.W522C b.H994R	a. 41.6% b. n.a.	CID-G/AI	Henoch schonlein purpura	Severe/ multiorgan (CNS vasculitis, stroke)	2.5 yrs	Serology Imaging	Anti-IFN-α/ω, anti-IL12, Coombs+ p-ANCA	AIHA	Steroids, IVIG	–	HSCT (MUD)	Good	Deceased, 6 yrs(stroke)	Unpublished
Patient 2	2.7 yrs	*RAG1*	a.R396C b.M435V	a. 0.6% b. 23.6%	CID-G/AI	Non-granulomatous-small vessel vasculitis	Severe/ multiorgan (digital necrosis)	1.5 yrs	Serology Imaging Biopsy	Anti-IFN-α/ω, anti-IL12, Coombs+	AIHA	Steroids	Cyclophosphamide. rituximab	HSCT (MUD)	Poor	Deceased, 2.7 yrs (idiopathic pneumonia syndrome)	Unpublished
Patient 3	48 yrs	*RAG1*	a.M1V b.R737H	a. n.a. b. 0.2%	CID-G/AI	Leukocytoclastic vasculitis	Mild/skin only	8 yrs	Serology Imaging Biopsy	Anti-IFN-α, ANA, anti-dsDNA, RF, anti-TG/TPO/TSHR	None	Steroids, IVIG	–	–	Good	Deceased, 48 yrs (COPD)	(1)
Patient 4	2 yrs	*RAG1*	a.R841Q b.F974L	a. 0% b. 56.5%	CID-G/AI	Non-granulomatous-small vessel vasculitis	Severe/ multiorgan (digital necrosis)	0.5 yrs	Serology Imaging	APLA, Coombs+, anti-platelet, anti-TPO	AIHA, ITP, AN, inflamatory myopathy, AIH	Steroids, IVIG	Rituximab	–	Good	Deceased, 2 yrs (enterobacter sepsis)	(6)
Patient 5	3.4 yrs	*RAG2*	a.G35A b.A456D	a.22.1% b. n.a.	CID-G/AI	n.a.	Severe/ multiorgan (n.a.)	0.5 yrs	n.a.	–	ITP	Steroids, IVIG	Alemtuzumab	HSCT (n.a.)	Good	Alive	Unpublished
Patient 6	15 yrs	*RAG1*	a.fs188X b.A444V	a. 2.7% b.1.4%	CID-G/AI	Non-granulomatous-small vessel vasculitis	Mild/ skin only	12.5 yrs	Serology Imaging Biopsy	–	Cutaneous granulomatosis	Steroids, IVIG	–	–	Good	Deceased, 15 yrs (pulmonary fibrosis)	(26)
Patient 7	5 yrs	*RAG1*	a.b.A444V	a.b. 1.4%	CID-G/AI	Kawasaki disease	Mild/ skin only	1.5 yrs	Serology	Anti-IFN-α, ANA	Macrophage activation syndrome (MAS), SLE	Steroids	–	–	Good	Alive	(28)
Patient 8	7.5 yrs	*RAG1*	a.b.R699W	a.b. 19.3%	CID-G/AI	Polyarteritis nodosa	Severe/ multiorgan (digital necrosis)	2.5 yrs	Serology Biopsy	–	AIHA	Steroids	Cyclophosphamide. azathioprine	–	Partial	Alive	(27)

## Results

### Demographics and Clinical Characterization of Patients With Vasculitis due to RAG Deficiency

**Figure 1:**
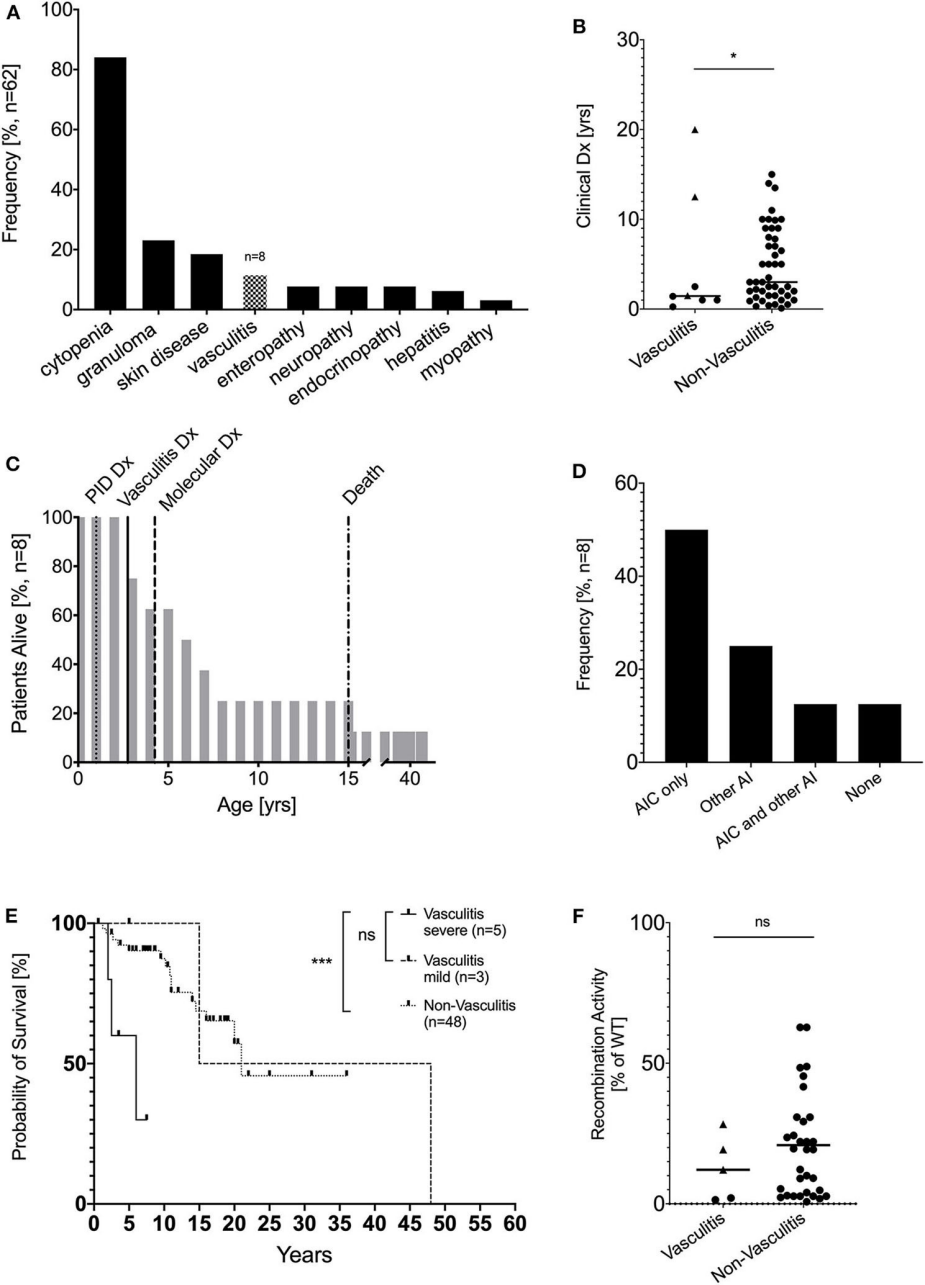
Demographic and clinical characterization patients with vasculitis due to RAG deficiency. **(A)** Vasculitis is the fourth most common complication of pRD with immune dysregulation in a cohort of 62 patients (modified from 13) **(B)** Clinical diagnosis of PID in years compared between patients with pRD (*n* = 8, circles = severe/multiorgan, rectangle = mild/skin-only) or without (*n* = 54) vasculitis and RAG deficiency (*p < 0.05) **(C)** Percent of patients alive by age and annotate clinical milestones **(D)** Kaplan-Meier curves comparing survival of RAG-deficient patients with (*n* = 8, dotted line) and without (*n* = 54, straight line) vasculitis **(E)** Overall frequency of autoimmune complications besides vasculitis in adult patients with RAG deficiency, AIC… autoimmune cytopenia, AI… autoimmunity **(F)** Recombination activity from all available RAG1/2 alleles (average of % wild-type protein). For the non-vasculitis control group only patients with CID-G/AI and AS phenotype were considered. (circles = severe/multiorgan, rectangle = mild/skin-only) (ns statistically not significant, *p ≤ 0.05, ***p ≤ 0.001).

In our cohort of 62 patients with hypomorphic RAG variants and autoimmune and/or hyperinflammatory complications, we identified 8 patients (12.9%) with episodes of vasculitis (Figure 1A). There was equal distribution of female (*n* = 4) and male patients (*n* = 4). The designated clinical phenotype was combined immunodeficiency with granuloma and/or autoimmunity (CID-G/A) in all 8 RAG deficient patients with vasculitis based on criteria described above (22). Patients with severe/multiorgan vasculitis were diagnosed with PID early in life (*n* = 5, median age of 1 years; age range of 0.25–2.5 years), in contrast patients with mild/ skin manifestation (*n* = 3, median age 12.5 years; age range 5–20 years, *p* = ns) and those without vasculitis (*n* = 48, median age 3 years, age range 0–15 years, *p* = 0.0171) were diagnosed later in life (Figure 1B). In 4 of 8 patients, vasculitis was the first clinical signs of immune dysregulation. The median duration of vasculitis episodes was 1.25 years, with no significant difference between severe/multiorgan and mild/skin manifestations (data not shown). Genetic diagnosis of underlying RAG deficiency was obtained at the median age of 4.25 years (range: 1.5–46 years) (Figure 1C). Besides development of vasculitis, the majority (*n* = 6) of the patients developed autoimmune complications. Cytopenia was the most common autoimmune complication, being present in 50% of the patients in our cohort, similar to other recently reported cohorts (21–77%) (13). Systemic autoimmunity/inflammatory conditions were observed in three patients, including inflammatory myopathy, cutaneous granulomatosis, and macrophage activation syndrome (MAS), and systemic lupus erythematodes (SLE). Only one patient developed no additional autoimmune complications besides vasculitis (Figure 1D).

The course of the disease was complicated by significant end organ damage, which was associated with a high mortality rate of 62.5% (5 of 8 patients) and a significantly reduced (*p* = 0.0436) median survival of 15 years compared to non-vasculitis pRD patients with immune dysregulation who had a median survival of 21.1 years. Although not significant, patients with severe/multiorgan vasculitis had overall reduced median survival of 6 years compared to patients with mild/skin limited vasculitis with a median survival of 15 years (Figure 1E). Leading causes of death in RAG patients with vasculitis included respiratory failure (idiopathic pneumonia syndrome post HSCT, pulmonary fibrosis, chronic obstructive pulmonary disease (*n* = 3), followed by sepsis with multi-organ failure (enterobacter sepsis, *n* = 1) and stroke due to central nervous system (CNS) vasculitis (*n* = 1) (Table 1).

The majority (*n* = 7) of the patients carried pathogenic *RAG1* variants, while one patient was compound heterozygous for pathogenic *RAG2* variants. To our knowledge, this is the first reported case of RAG2 deficiency and vasculitis. There was no significant difference in the relative recombinase activity level between RAG variants presenting with or without vasculitis and between severe and mild vasculitis manifestation (Figure 1F).

### Detailed Clinical Description of Vasculitis and Treatment Outcome in Patients With RAG Deficiency

**Figure 2:**
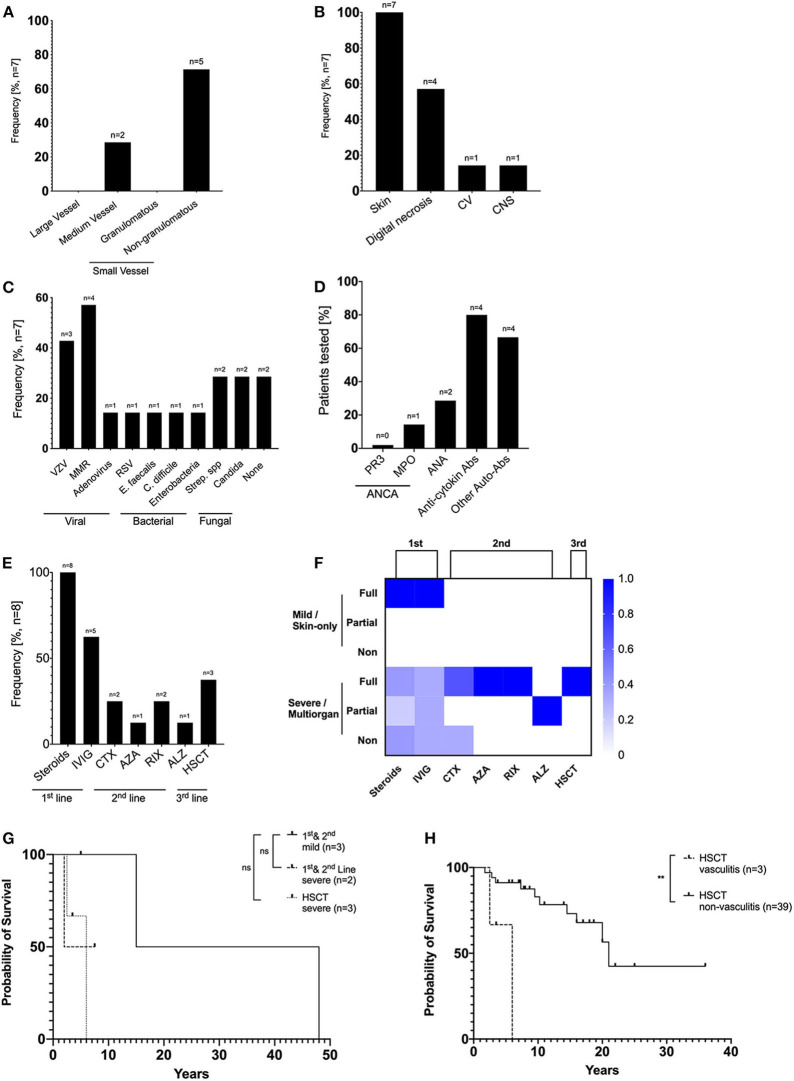
Detailed description of vasculitis and treatment outcome in patients with RAG deficiency. **(A)** Relative frequency of large, medium and small vasculitis among RAG patients **(B)** Relative frequency of target organs affected by vasculitis **(C)** Relative frequency of potential infectious trigger preceding episodes of vasculitis. **(D)** Prevalence of autoantibodies among RAG patients. Anti-cytokine included antibodies against IFN α, ω, and IL-12. Other autoantibodies included anti-erythrocyte, platelet, dsDNA, TPO, TG, ALPA antibodies. **(E)** Treatment strategies used in patients with vasculitis due to RAG deficiency **(F)** Treatment response scored for first line (steroids ± IVIG), second line (biologicals or immunosuppressives), or third line (Haematopoietic stem cell transplantation) was scored using the following criteria: “non,” no clinical response or side effects were limiting; “partial,” clinical improvement but therapeutic escalation was required; or “full,” clinical improvement with no escalation. **(G)** Kaplan-Meier curves comparing survival of RAG-deficient patients with severe, systemic multiorgan vasculitis that underwent HSCT (*n* = 3, dashed line), patients with severe, systemic multiorgan vasculitis that received first/second line therapy (*n* = 2, dotted line) and patients with mild, skin limited vasculitis (*n* = 3, straight line). **(H)** Kaplan-Meier curves comparing survival of RAG-deficient patients with vasculitis (*n* = 3, dashed line) and RAG-deficient patients without vasculitis (*n* = 39, straight line) that underwent HSCT (ns statistically not significant, **p ≤ 0.01).

Childhood vasculitis is classified based on vessel size, including large, medium, and small vessel vasculitis (30). Detailed clinical information of vasculitis was available in 7 patients with RAG deficiency. In our cohort, we observed predominately non-granulomatous-small vessel vasculitis (*n* = 5), including one case of Henoch-Schönlein purpura (IgA vasculitis), one case of cutaneous leukocytoclastic vasculitis and 3 cases of unspecified non-granulomatous-small vessel vasculitis. Two patients displayed medium vessel vasculitis, one case of childhood polyarteritis nodosa and one case of Kawasaki disease. There were no cases of large vessel nor granulomatous-small vessel vasculitis identified (Figure 2A). Vasculitis was diagnosed based on clinical history, serology, imaging, and/or biopsy (Table 1). The disease was complicated by severe end organ complications. In particular, skin involvement was seen in all seven patients, and digital necrosis in four CNS vasculitis and cardiovascular complications were seen in one patient each (Figure 2B).

Vasculitis may develop as a result of infectious or non-infectious triggers (31). We therefore tried to correlate potential infectious triggers with the onset of vasculitis. Five patients developed vasculitis following viral infection (varicella zoster virus, adenovirus, or respiratory syncytial virus) or administration of attenuated vaccine (measles, mumps, and rubella), four patients had bacterial infections (*E. faecalis, C. difficile, Enterobacteria, Streptococcus spp*.), two patients had fungal infection (Candida). No correlation of a potential infectious trigger and development of vasculitis could be identified in two patients (Figure 2C). The median duration of time elapsed from viral infections or vaccination to the development of vasculitis was 5 months (range 0–9 months, *n* = 5) (data not shown).

In addition, we analyzed if autoantibodies associated with systemic vasculitis can be used as a diagnostic biomarker in RAG deficient patients. The majority of the patients were anti-neutrophil cytoplasmic antibodies (ANCA) and antinuclear antibodies (ANA) negative (ANCA-negative: 5/6; ANA-negative: 5/7). However, 4 out of 5 patients were positive for anti-cytokine antibodies (targeting IFN-α, -ω, and IL-12), confirming what has been described in previous reports (29) (Figure 2D).

Topical and systemic steroids [± immunoglobulin replacement therapy (IgRT)] were used in all patients as first line therapy and were sufficient to induce remission of vasculitis limited to skin manifestations in three patients. First (steroids, IVIG) and second-line treatment (cyclophosphamide, azathioprine rituximab, and alemtuzumab) had limited effectiveness in four patients with severe, systemic multiorgan complications. Three patients were referred for HSCT as third line therapeutic approach, leading to remission of vasculitis in all of them (Figures 2E,F). Comparisons of overall survival between first/second line treatment and HSCT revealed no statistically significant difference. Patients with severe/multiorgan complications that underwent HSCT (*n* = 3) had a median survival of 6 years, whereas patients with severe complications that received first/second line treatment (*n* = 2) had a median survival of 7 years. Patients with mild, skin-limited vasculitis (*n* = 3) had a median survival of 15 years (Figure 2G). RAG deficient patients with vasculitis (median survival 6 years) that were treated with HSCT had an overall worse outcome than patients without vasculitis that underwent HSCT (median survival 21 years, *p* = 0,0018) (Figure 2H).

### Phenotypic Description of RAG Deficient Patients With Vasculitis

**Figure 3:**
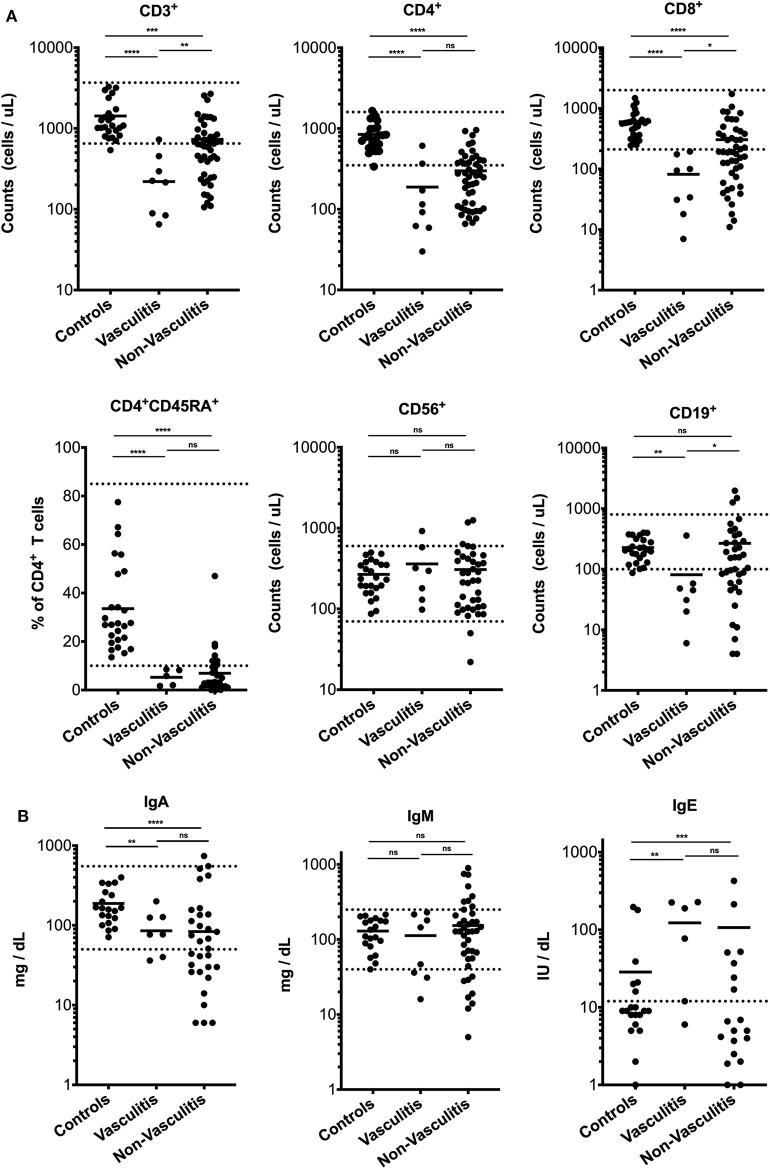
Lymphocyte cell counts and serum immunoglobulins in patients with vasculitis due to RAG deficiency. **(A)** CD3^+^ T cells, CD4^+^ T cells, naïve CD4^+^CD45RA^+^ T cells, CD8^+^ T cells, CD56^+^ NK cells, and CD19^+^ B cells of age-matched healthy controls (*n* = 25), RAG deficient patients with (*n* = 8) without (*n* = 46) vasculitis **(B)** Immunoglobulin titers of age-matched healthy controls (*n* = 25) RAG deficient patients with (*n* = 8) without (*n* = 46) vasculitis. Dashed line represents normal values. Statistically significant differences obtained in intergroup comparisons were confirmed by Mann Whitney *U*-test. Values of *p* < 0.05 were considered as significant (ns statistically not significant, *p ≤ 0.05, **p ≤ 0.01, ***p ≤ 0.001, ****p ≤ 0.0001).

Next, we compared the immunologic phenotype of RAG deficient patients with vasculitis to those without vasculitis and healthy pediatric and adult controls. The dominant laboratory feature among patients with vasculitis associated with pRD was a severe T cell lymphopenia [mean T cell count: 220 cells/μl (range 65–727), *p* = 0.0073]. In comparison, T cell lymphopenia was less pronounced in patients with pRD and immune dysregulation but without vasculitis (mean T cell count: 635 cells/μl; range 106–2,678). We observed a trend toward lower counts of CD4^+^ T cells (mean: 104 cells/μl; range: 30–611, *p* = 0.0577) and of naïve CD4^+^ T cells (mean: 5 cells/μl; range 1.69–8.5, *p* = 0.0991) in vasculitis patients than in the non-vasculitis group (CD4^+^ T cells, mean: 257 cells/μl; range 66–958; naïve CD4^+^ T cells, mean: 7 cells/μl; range 0.04–47), although this did not reach significance. Interestingly, all patients with vasculitis were severely CD8^+^ T cell lymphopenic with a mean of 81 cells/μl (range 7–194, *p* = 0.0116) compared to the non-vasculitis group (mean CD8^+^ cells count: 304 cells/μl; range 11–1,731). B cell counts were variably low compared to controls with a mean of 81 cells/μl (range 6–359). There was no significant difference in NK cell numbers between different groups (Figure 3A). T cell proliferation to phytohemagglutinin (PHA) was comparable between patients with vasculitis (in average 25,601 cpm) and patients without vasculitis (in average 21,000 cpm) (data not shown). IgG levels could not be assessed because the majority of the patients were on IgRT, and no native IgG levels were recorded. IgA (85.2 mg/dl, 0–200) and IgM (112.6 mg/dl, 16–230) serum levels were highly variable, and no significant difference to non-vasculitis pRD patients could be observed. Elevated IgE was detected in 4/6 of cases (Figure 3B).

## Discussion

In the last decade, the spectrum of PIDs has extended from being defined by a susceptibility to infections alone to include features of immune dysregulation (29). A recent study of the French national PID registry observed a wide range of autoimmune and autoinflammatory complication (26.2%) among PID patients (32). All types of PIDs were associated with a risk to develop immune dysregulation, although T-cell PIDs and CVID appear to have the greatest risk (32). Among PIDs with CID with immune dysregulation, partial RAG deficiency is one of the most common entities (33, 34). Initially found to account for T- and B-cell-negative SCID, pathogenic RAG *gene* variants have been subsequently associated with a broader spectrum of phenotypes, including autoimmunity and immune dysregulation (13).

Herein, we have presented vasculitis as key component of morbidity among patients with hypomorphic *RAG* variants. Vasculitis was associated with a high mortality rate of 62.5% and a reduced median survival of 15 years. Although RAG-mutated patients with vasculitis were recognized earlier than those without vasculitis, their overall survival and life expectancy were severely reduced, confirming that autoimmunity worsens the prognosis in patients with PIDs. Treatment strategies need to be carefully examined to balance the efficacy and toxicity of biologic and non-biologic immunosuppressive drugs in RAG deficient patients.

Abnormalities of central and peripheral T and B cell tolerance play key mechanisms in immune dysregulation in patients with hypomorphic RAG variants. Central B cell tolerance is affected by a failure to reexpress the RAG complex during receptor editing of immature B cells in the bone marrow (35). Peripheral B cell tolerance is disturbed by an inability to deplete anergic self-reactive B cells due to survival in a milieu with increased BAFF levels (36, 37). Impaired B cell tolerance in RAG deficiency is highlighted by a wide spectrum of serum autoantibodies, including neutralizing antibodies against interferon-α, interferon-ω, and IL12 observed in our cohort. The majority of RAG deficient patients with vasculitis were positive for anti-cytokine antibodies, which were demonstrated to aggravate immune dysregulation, hyperinflammation with increased type-1 interferon signature and increased susceptibility to prolonged viral infection (29). As an example, for hyperinflammation in the setting of infections, it has been described for vaccine-derived rubella in cutaneous granuloma formation in RAG deficient patients (38). While we tried to correlate the development of vasculitis with potential infectious trigger, further research needs to be done to identify a causative trigger.

Recent studies have identified vasculitis as an uncommon complication of PIDs, having been observed in 1–1.6% of the patients reported in the French national PID registry and in the USIDNET registry (32, 39). In contrast, we identified vasculitis to be a prevalent complication among patients with hypomorphic *RAG* variants and immune dysregulation (12.9%). Similar to RAG deficiency, there are other PIDs specifically associated with vasculitis. The differential diagnosis should include ADA2 deficiency (40), CVID (38%) Wiskott-Aldrich syndrome (WAS) (26%) (39). Unlike in ADA2 deficiency, where stroke is predominant, in our cohort of 8 patients, only one patient had a stroke.

We have recently reported that pathogenic variants in the *RAG* genes can result in significant phenotypic variability, and may occur in 1 in 500 patients with antibody deficiency, including CVID (41). We therefore recommend that partial RAG deficiency should be considered for patients with antibody deficiency and vasculitis, especially when associated with other autoimmune manifestations, and/or progressive T cell lymphopenia. Autoantibodies that are frequently associated with typical forms of vasculitis may be lacking in patients with hypomorphic *RAG* variants, as indicated by the fact that the majority of patients presented with ANCA negative small vessel vasculitis. Therefore, conventional vasculitis autoantibody panel should be extended to test for antibodies targeting cytokines, and in particular IFN-α, -ω, and IL-12 (29).

Given the importance of providing optimal care for patients with PIDs, further prospective studies are needed to identify potential pathogenic mechanisms and help guide in the development of optimal treatment of vasculitis in patients with RAG deficiency.

## Data Availability Statement

All datasets generated for this study are included in the article/**Supplementary Material**.

## Ethics Statement

Clinical data were collected, and research laboratory studies were performed on de-identified samples under IRB approved protocols at University of South Florida (IRB protocol #Pro00025693 at University of South Florida for JW). Written informed consent to participate in this study was provided by the participants' legal guardian/next of kin.

## Author Contributions

CG conceived the presented idea, interpreted and analyzed the results, and wrote the manuscript. JF, ZF, BU, and JS curated the patient database, assisted with data interpretation performed functional assays. JWS, SP, MD, S-YP, LH, MH, BN, DM, SS, SM, PY, EN, SÖ, KB, JF, and HW provided clinical data. ME, LN, and LC reviewed the clinical information presented. JW encouraged to describe this cohort and clinical course in the context of internationally accepted guidelines and supervised the findings of this work. All authors discussed the results and contributed and agreed to the final manuscript.

## Conflict of Interest

ME was employed by Biomedizinische Forschungs GmbH. The remaining authors declare that the research was conducted in the absence of any commercial or financial relationships that could be construed as a potential conflict of interest.
